# The kinetic characteristics of human and trypanosomatid phosphofructokinases for the reverse reaction

**DOI:** 10.1042/BCJ20180635

**Published:** 2019-01-18

**Authors:** Peter M. Fernandes, James Kinkead, Iain W. McNae, Frédéric Bringaud, Paul A.M. Michels, Malcolm D. Walkinshaw

**Affiliations:** 1 School of Biological Sciences, University of Edinburgh, Michael Swann Building, Max Born Crescent, Edinburgh EH9 3BF, U.K.; 2 Laboratoire de Microbiologie Fondamentale et Pathogénicité (MFP), Université de Bordeaux, CNRS UMR-5234, Bordeaux, France

**Keywords:** human, *Leishmania*, phosphofructokinase, reverse reaction, *Trypanosoma cruzi*, *Trypanosoma brucei*

## Abstract

Eukaryotic ATP-dependent phosphofructokinases (PFKs) are often considered unidirectional enzymes catalysing the transfer of a phospho moiety from ATP to fructose 6-phosphate to produce ADP and fructose 1,6-bisphosphate. The reverse reaction is not generally considered to occur under normal conditions and has never been demonstrated for any eukaryotic ATP-dependent PFKs, though it does occur in inorganic pyrophosphate-dependent PFKs and has been experimentally shown for bacterial ATP-dependent PFKs. The evidence is provided via two orthogonal assays that all three human PFK isoforms can catalyse the reverse reaction *in vitro*, allowing determination of kinetic properties. Additionally, the reverse reaction was shown possible for PFKs from three clinically important trypanosomatids; these enzymes are contained within glycosomes *in vivo*. This compartmentalisation may facilitate reversal, given the potential for trypanosomatids to have an altered ATP/ADP ratio in glycosomes compared with the cytosol. The kinetic properties of each trypanosomatid PFK were determined, including the response to natural and artificial modulators of enzyme activity. The possible physiological relevance of the reverse reaction in trypanosomatid and human PFKs is discussed.

## Introduction

Phosphofructokinase (PFK) catalyses the phosphorylation of fructose 6-phosphate (F6P) to fructose 1,6-bisphosphate (F1,6BP) and plays a central role in the glycolytic pathway of prokaryotes and eukaryotes. The enzymatic step catalysed by PFK is conserved in most organisms from Eukarya, Bacteria and Archaea. Despite an enzyme mechanism that has been conserved for over 2 billion years, different PFK families have evolved interestingly diverse regulatory mechanisms associated with considerable differences in protein sequence and architecture. There is increasing interest in PFK as a drug target in human diseases, including diabetes [1] and cancer [2,3]. Additionally, glycolysis is a valid therapeutic target for killing pathogens that rely exclusively on glucose catabolism for their ATP supply; previous work has shown the effectiveness of this approach against trypanosomatid parasites [4]. PFKs from the three trypanosomatid species that cause significant mortality and morbidity: *Trypanosoma brucei* (Human African Trypanosomiasis), *Trypanosoma cruzi* (Chagas disease) and *Leishmania* spp*.* (Leishmaniasis) are all potential drug targets.

Two main evolutionary groups of PFK are distinguished by their phospho-donor substrates. The pyrophosphate-dependent group uses inorganic pyrophosphate (PPi) as the phospho-donor and is found in plants and certain protists, including amoeba and bacteria. The second group uses ATP as the phospho-donor and is found in many other bacteria and protists, plants and all vertebrates (note that plants usually contain PFKs from both groups). Phylogenetic and structural analyses demonstrate that the two groups evolved from a common ancestor, though amino acid sequence identities are low (∼25%) [5]. Despite these differences, there are similarities in catalytic mechanism; in one interesting case the PPi–PFK of *Entamoeba histolytica*, which has a 10^6^-fold preference for PPi over ATP, could be converted to an ATP-dependent enzyme by a single mutation [6]. This supports the idea that the ATP-dependent PFKs are the primordial form from which, on multiple occasions, the PPi-dependent enzymes evolved. However, the evolutionary path is complex, with amino acid sequence comparisons suggesting the ATP-dependent PFK found in trypanosomatids developed through an ancestral PPi reliant stage before switching back to ATP as a substrate [7,8].

A biochemically important distinction between the two families is that all PPi-PFKs readily carry out both the forward and reverse enzyme reactions under physiological conditions. The biological consequence is that organisms that use PPi-dependent PFKs, for the most part, do not require fructose-1,6-bisphosphatase enzymes to carry out the reverse dephosphorylation step required in the gluconeogenic pathway. Possibly because of its ability to carry out the reaction in both directions, the PPi–PFK family shows little evidence of allosteric regulatory mechanisms controlling enzyme activity, though this is not universal [9]. In contrast, ATP-dependent PFKs have evolved a wide range of allosteric mechanisms, with associated differences in protein chain lengths and architecture.

The active form of bacterial ATP-dependent PFKs is a homo-tetramer with subunits of ∼35 kDa. In yeasts, a gene duplication/fusion event yielded double-size chains and these subsequently underwent additional duplications to give an octamer comprised of homologous catalytic and regulatory subunits each with a molecular mass of 110–120 kDa [10]. Mammals have tetrameric PFKs, with subunits of 85 kDa. The N-terminal half of the double enzyme was constrained to retain the catalytic function, while the substrate-binding sites of the C-terminal half evolved into effector-binding sites [11]. Higher levels of regulation in mammals are also achieved by three distinct isoforms with varying properties (denoted as PFK-M, PFK-L and PFK-P) which are expressed in a tissue-specific pattern [12]. Trypanosomatid PFKs are intermediate in size (∼55 kDa); these are strictly ATP dependent but have amino acid sequences closer to the PPi family and can, therefore, be regarded as chimaeras. X-ray structures of trypanosomatid PFKs show major differences compared with other ATP-dependent PFKs; in particular, the C-terminal extensions can form long helices, acting as reaching arms to hold the tetramer together [8].

Structural differences between ATP-dependent PFKs derive from varying requirements for allosteric regulation. The smaller bacterial ATP-dependent PFKs are activated by ADP and GDP alone [13] with *Escherichia coli* PFK used in a definitive study by Monod and co-workers to support the now classic allosteric model of enzyme kinetics [14]. In trypanosomatid PFKs, AMP is the only known activator, while in human PFKs the non-catalytic C-domain of each isoform binds the allosteric activators AMP, ADP and fructose 2,6-bisphosphate (F26BP) [15].

The evolution of these tightly regulated allosteric effector systems in the ATP-dependent PFK family contrasts with the less regulated bi-directional activity of the PPi-dependent family. For the ATP-dependent PFKs the forward enzymatic reaction is favoured under physiological conditions, often being regarded as an essentially irreversible reaction under normal conditions. Indeed, the reverse reaction (F16BP+ADP⇆F6P+ATP) has never been demonstrated for any eukaryotic ATP-dependent PFK, including any of the human isoforms, *in vitro* or *in vivo*. In the present paper, we demonstrate that the reverse reaction is possible under experimental conditions for all three human PFK isoforms and the three trypanosomatid PFK orthologues, and present the kinetic properties for these reactions. The results suggest that the reverse reaction could occur under certain physiological conditions.

## Methods

**Figure 1.**
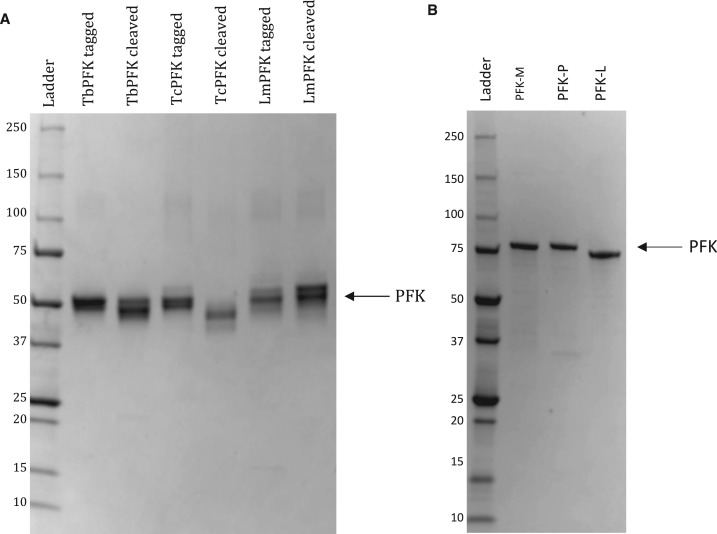
Purification of bacterially-expressed trypanosomatid and human PFKs. SDS–PAGE gels (4–20%) showing (**A**) trypanosomatid PFKs were produced at high purity but the removal of tags was only partially successful and (**B**) human PFK isoforms were produced at high purity but with the anomalous migration of PFK-L. Tagged enzymes were used for all experiments due to incomplete tag cleavage and reduction in enzyme activity after cleavage (data not shown), likely secondary to conditions required to remove tag. Ladder markers are in kDa.

The identity of all recombinant PFKs was confirmed by SDS–PAGE showing highly pure PFKs of expected molecular mass (Figure 1). Additionally, western blots and MALDI-TOF mass spectrometry using a Bruker Ultraflex instrument confirmed protein identities (data not shown).

### Production of trypanosomatid PFKs

N-terminally His_6_-tagged trypanosomatid PFK DNA sequences, codon optimised for *E. coli* expression, were inserted into pET28a or pDEST17 expression plasmids. The recombinant plasmids were used to transform chemically competent *E. coli* cells which were grown on LB agar plates with corresponding antibiotic (Table 1). Single colonies were inoculated into 500 ml media in 2 l conical flasks and grown in a shaking incubator at 250 rpm and 37°C to an OD_600nm_ 0.8–0.9, then cold shocked at 4°C for 30 min. PFK expression was induced with 1 mM isopropyl β-d-1-thiogalactopyranoside (IPTG) for 16 h at 100 rpm and 18°C before harvesting the cells via centrifugation and removal of supernatant.

**Table 1:** Expression conditions for trypanosomatid PFKs in *E. coli*

	DNA source strain	Expression plasmid	Expression cell line	Antibiotic	Media
*T. b. brucei*	Lister 427	pET28a	C41 (DE3)	Kanamycin	2xYT broth
*T. cruzi*	CL Brener	pET28a	BL21 (DE3)	Kanamycin	Superbroth
*L. infantum*	JPCM5	pDEST17	C41 (DE3)	Carbenicillin	LB broth

Cell pellets from 1 l cultures were suspended in 50 ml lysis buffer [50 mM triethanolamine (TEA), 5 mM MgCl_2_, 50 mM KCl, 10% glycerol, pH 7.4], supplemented with Roche cOmplete™ EDTA-free Protease Inhibitor Cocktail and ∼5 mg bovine pancreas deoxyribonuclease (Sigma–Aldrich D5025) and lysed with Constant Cell Disruption Systems at 25kPsi and centrifuged. Filtered supernatant was loaded onto a cobalt-charged HiTrap 1 ml FF immobilised metal affinity chromatography (IMAC) column (GE Healthcare) equilibrated in wash buffer (50 mM TEA, 300 mM NaCl, 20 mM imidazole, 10% glycerol, pH 8.0) in a GE Healthcare ÄKTA purifier system at 6°C. Impurities were removed by further wash buffer steps with gradually increasing imidazole concentrations before PFK eluted with elution buffer (50 mM TEA, 300 mM NaCl, 500 mM imidazole, 10% glycerol, pH 8.0). *T. brucei* and *T. cruzi* PFK (TbPFK and TcPFK) eluates were loaded onto a HiPrep Sephacryl™ S-200 16/60 column (GE Healthcare), pre-equilibrated with gel filtration buffer (20 mM TEA, 5 mM MgCl_2_, 50 mM KCl, 10% glycerol, pH 7.4) and tetrameric fractions eluted with 1.5 column volumes (CVs) of gel filtration buffer. *Leishmania infantum* PFK (LmPFK) IMAC eluates were loaded onto a HiPrep 26/10 Desalting column (GE Healthcare) pre-equilibrated with gel filtration buffer and eluted using 1.5 CV gel filtration buffer. Samples were concentrated to 1 mg/ml with Vivaspin® 20 ml 30 000 kDa Molecular Weight Cut-Off (MWCO) spin concentrators. Aliquots were flash-frozen and stored at −80°C until required. Tag removal was attempted but only partially successful (Figure 1A); tagged protein was therefore used.

### Production of human PFKs

Plasmid pJJH71 [16] containing yeast codon optimised cDNA for His_6_-tagged PFK-M1, PFK-L1 or PFK-P1 was used to transform PFK-deficient *Saccharomyces cerevisiae* [16] via electroporation which was subsequently grown on YPDA (yeast extract, peptone, dextrose, adenine broth) agar plates. Colonies were transferred to 2 l conical flasks containing 500 ml YPDA medium with 50 µ/ml carbenicillin and cultures grown using an Infors HT Multitron standard shaking incubator at 30°C and 250 rpm before harvesting the yeast via centrifugation and removal of supernatant.

Cell pellets from 2 l cultures were suspended in lysis buffer [50 mM TEA, 300 mM KCl, 10 mM imidazole, 1 mM TCEP (tris(2-carboxyethyl)phosphine), 1 mM ATP/F6P] supplemented with Roche cOmplete™ EDTA-free Protease Inhibitor Cocktail and ∼5 mg bovine pancreas deoxyribonuclease at 8% w/v and lysed with Constant Cell Disruption Systems at 40 kPsi and centrifuged. Filtered supernatant was loaded onto a nickel-charged HiTrap 1 ml FF IMAC column equilibrated in wash buffer (50 mM TEA, 300 mM KCl, 10 mM imidazole, 1 mM TCEP, 1 mM ATP/F6P, 10% glycerol) in an ÄKTA purifier system at 6°C. Impurities were removed by further wash buffer steps with gradually increasing imidazole concentrations before PFK eluted with elution buffer (50 mM TEA, 300 mM KCl, 500 mM imidazole, 1 mM TCEP, 1 mM ATP/F6P). PFK-M was further purified using a GE Healthcare HiPrep Sephacryl S300 16/600 size-exclusion chromatography column pre-equilibrated with gel filtration buffer (50 mM TEA, 500 mM KCl, 5 mM MgCl_2_, 1 mM TCEP, 1 mM ATP/F6P, 10% glycerol). For PFK-L and PFK-P a GE Healthcare Superose 6 10/300 size-exclusion column was used. Samples corresponding to tetrameric protein (340 kDa) were pooled and concentrated using a pre-equilibrated 20 ml 30 000 kDa MWCO spin concentrator to above 0.3 mg/ml. Aliquots were flash-frozen and stored at −80°C until required. All buffers were at pH 8, except for PFK-M purifications (pH 7.4).

### Demonstration of reverse reaction using an endpoint assay

The Promega Kinase-Glo ATP assay system (V6713) was used to measure ATP production in the reverse PFK reaction. The energy of the ATP produced was converted to light via the luciferase/luciferin reaction in an endpoint reaction. Ten microlitres of PFK at 2 μg/ml was added to 100 μl assay buffer (50 mM TEA, 10 mM MgCl_2_, 0.1% w/v BSA, 0.005% TWEEN20, 1% DMSO, pH 7.4) containing 5 mM ADP in a white non-binding 96-well plate. Incubation was carried out at 4°C for 20 min, followed by 10 min at room temperature. F6P was added to a final concentration of 5 mM and the plate was then centrifuged at 1000 rpm for 30 s, and further incubated at room temperature for 60 min. Twenty-five microlitres of the ‘Kinase-Glo reagent' was added to each well for a final incubation period of 30 min. Assay output was measured as luminescence using a Molecular Devices Spectramax M5 Multi-Mode Plate Reader and converted to ATP concentrations using control data from a constructed ATP titration curve.

### Determination of kinetic characteristics using an enzyme-linked kinetic assay

Conversion of F16BP (Sigma F6803) and ADP (Sigma A4386) to F6P and ATP was measured using an enzyme-linked assay. F6P was converted to glucose 6-phosphate (G6P) by phosphoglucose isomerase (PGI), and subsequently to 6-phosphogluconolactone by glucose-6-phosphate dehydrogenase (G6PD), with concurrent reduction of NAD^+^ to NADH. Formation of NADH was measured via absorbance of UV at 340 nm. ATP produced was re-converted into ADP by glycerol kinase (GK) (in the presence of glycerol) to keep the ATP/ADP ratio low.

Fifteen microlitres of assay buffer (50 mM TEA, 100 mM KCl, 10 mM MgCl_2_, 1 mM TCEP, 10% glycerol, pH 7.4) was added to 40 µl of assay mix [NAD^+^ (Sigma NAD100-RO), G6PD (Sigma G8529), PGI (Sigma P9544) and GK (Sigma G6142)], then 20 µl of ADP titration (or 15 mM ADP stock) in a clear 96-well plate. The plate was incubated at 25°C for 2 min. Five microlitres of 0.2 mg/ml PFK was added and the reaction initiated with 20 µl F16BP titration (or 25 mM F16BP stock). UV absorbance at 340 nm was measured at 13 s intervals for 15 min in a Molecular Devices Spectramax M5 Multi-Mode Plate Reader at 25°C.

Time-dependent absorbance change was converted into the rate of NADH oxidation (μM s^−1^) or specific activity (μmol min^−1^ mg^−1^) using the Beer-Lambert law (molar extinction coefficient of NADH 6.22 mM^−1^ cm^−1^). Reaction rates for each well were calculated using an 8-point (104 s) rolling average. Kinetic parameters of the steady-state stage of the reaction were determined with GraphPad Prism 7. Non-linear regression analysis was performed on substrate titration data, with curves fitted using allosteric sigmoidal models, enabling determination of kinetic values.

## Results

### Proof of concept for reversal of the direction of PFK reaction

**Figure 2.**
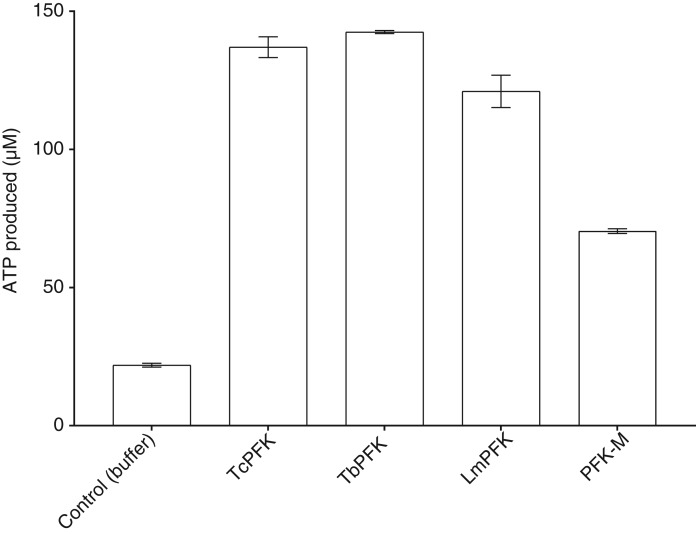
In vitro ATP synthesis by trypanosomatid PFKs and human PFK-M. ATP is produced by PFKs from ADP and F16BP using an endpoint assay (ADP 5 mM, F16BP 5 mM, error bars are standard deviations; *n* = 2).

Proof of concept for reversal of the direction of the PFK reaction was established with the ‘Kinase-Glo’® assay. Figure 2 shows that the addition of PFK to F16BP and ADP enabled much greater production of ATP than in control samples alone. Conversion from concentrations in µg/ml to molar concentrations shows that human and trypanosomatid PFKs produce similar amounts of ATP per mole. The yield of ATP from control experiments may derive from the spontaneous conversion of ADP into ATP occurring at high ADP concentrations (2ADP⇆ATP+AMP). Potential contamination of ADP stocks with ATP was prevented by using Ultrapure ADP (>99% purity; Promega). Minimal concentrations of ATP were present in PFK stocks, as shown by control experiments without F16BP or ADP; amounts were insufficient to confound results (data not shown).

### Kinetic properties for reverse activity by trypanosomatid PFKs

**Figure 3.**
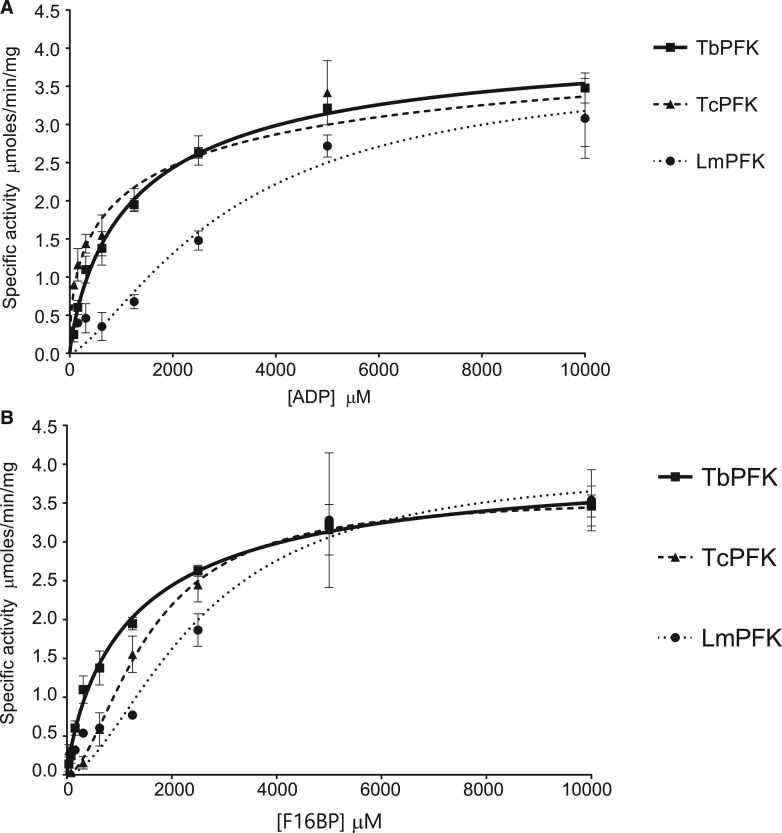
Kinetics of the reverse reactions by trypanosomatid PFKs. (**A**) Trypanosomatid PFKs have different kinetic responses for ADP titrations (F16BP 5 mM). (**B**) Trypanosomatid PFKs have different kinetic responses for F16BP titrations (ADP 3 mM). (Error bars are standard deviations, *n* = 3.)

An enzyme-linked assay was used to measure F6P production from the reverse reaction catalysed by PFK isoforms. Michaelis–Menten curves using allosteric sigmoidal models were generated for ADP titrations (Figure 3A) and F16BP titrations (Figure 3B). TbPFK is the most active isoform, with the lowest $K_{0.5}^{\mathrm{ADP}}$ and $K_{0.5}^{F16\mathrm{BP}}$ (*K*_0.5_ defined as the concentration of substrate at which half maximal enzyme velocity is reached, analogous to Michaelis–Menten constant [*K*_M_]). TcPFK has similar kinetic parameters to TbPFK. LmPFK has a slightly lower *V*_max_ and markedly lower affinities for both substrates, in keeping with known lower activities in the forward reaction [17]. Full kinetic parameters are listed in Table 2. Control experiments without PFK did not produce significant quantities of ATP (data not shown).

**Table 2:** Kinetic parameters for trypanosomatid PFKs in the reverse reaction Values are mean averages (±standard error of mean; *n* = 3). Large standard errors for TcPFK data result from varying degree of fit of non-linear regression sigmoidal models, despite narrow error bars apparent in Figure 4. h(F16BP) and h(ADP) are the Hill coefficients derived from fitting the Michaelis-Menten curves shown in Figure 3.

	*V*_max_ (µmol/min.mg)	$K_{0.5}^{ADP}$ (µM)	$K_{0.5}^{F16BP}$ (µM)	*h* (ADP)	*h* (F16BP)
TcPFK	4.77 (1.37)	1768 (2238)	1540 (153)	0.51 (0.14)	1.72 (0.22)
TbPFK	4.22 (0.28)	1382 (275)	1287 (202)	0.83 (0.07)	0.86 (0.06)
LmPFK	3.79 (0.69)	3137 (1084)	2495 (624)	1.42 (0.37)	1.71 (0.65)

Both TcPFK and TbPFK reverse reaction kinetics were consistent with allosteric sigmoidal models for F16BP titrations and for ADP titrations, with LmPFK reaction kinetics showing no statistical difference between the Michaelis–Menten and allosteric sigmoidal models; the allosteric sigmoidal model was preferred to retain consistency.

### The reverse PFK reaction can be allosterically modulated in trypanosomatid PFKs

AMP is a known activator, and sole physiological allosteric effector, of the forward PFK reaction in trypanosomatids [15]. The effect of AMP on the reverse activity of trypanosomatid PFKs was assessed using the enzyme-linked kinetic assay described above. All three trypanosomatid PFKs were activated in the presence of 0.5 mM AMP, with the $k_{\mathrm{cat}}/K_{0.5}^{F16\mathrm{BP}}$ value increasing 2-fold for TbPFK and TcPFK, and 10-fold for LmPFK (Supplementary Figures S1 and S2). F26BP, a potent activator of the forward reaction in many eukaryotic ATP-dependent PFKs (but not trypanosomatids) and bi-directional PPi-dependent plant PFKs [18], did not have any effect at 1 mM concentration.

A series of allosteric inhibitors deriving from a compound with anti-TbPFK activity [4] were tested for effects on the reverse PFK reaction catalysed by trypanosomatid PFKs. These compounds inhibited the reverse trypanosomatid PFK reaction with similar potencies to the forward reaction (results not shown).

### The reverse PFK reaction in human PFK isoforms

**Figure 4.**
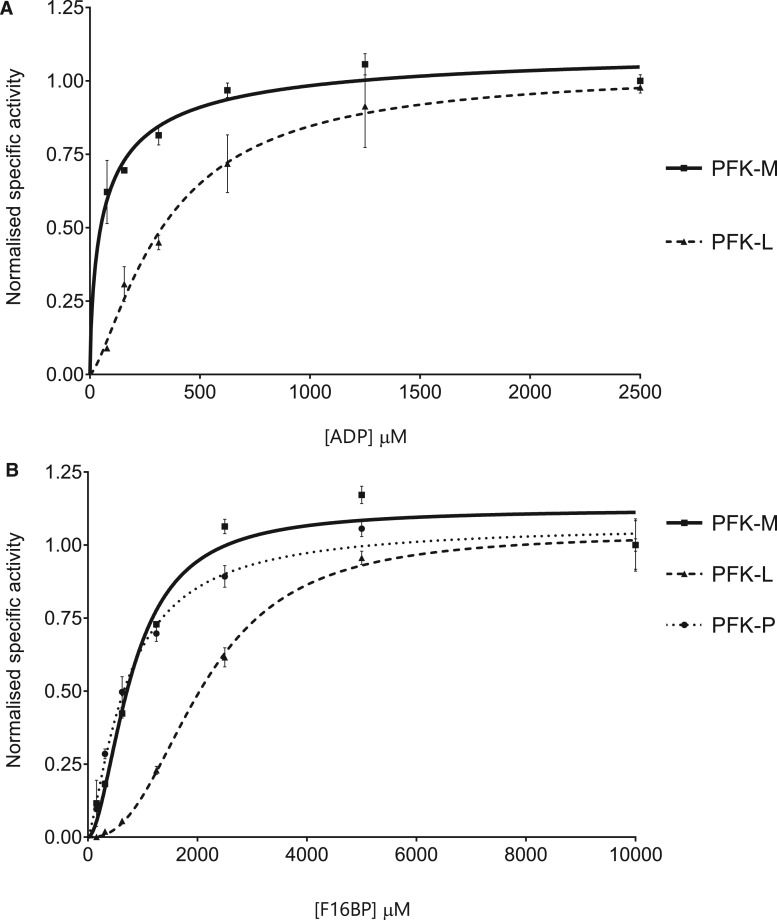
Kinetics of the reverse reactions by the human PFK isoforms. (**A**) Human PFK isoforms have different kinetic responses for ADP titrations (F16BP 5 mM). (**B**) Human PFK isoforms have different kinetic responses for F16BP titrations (ADP 3 mM). PFK-P data are missing for (**A**) due to lack of enzyme stability under these assay conditions. (Error bars are standard deviations, *n* = 3, isoforms were individually normalised to highest specific activity.)

Kinetic parameters for human PFK isoforms were determined using the enzyme-linked kinetic assay. Michaelis–Menten curves using allosteric sigmoidal models were generated for ADP titrations (Figure 4A) and F16BP titrations (Figure 4B). PFK-M and PFK-L have similar *V*_max_ values, but PFK-P is much less active. However, PFK-L has much lower affinities for ADP and F16BP than the other isoforms, with the highest $K_{0.5}^{\mathrm{ADP}}$ and $K_{0.5}^{F16\mathrm{BP}}$ ($K_{0.5}^{\mathrm{ADP}}$for PFK-P not determined). Kinetic parameters are listed in Table 3. Human PFK isoforms were purified in the presence of 1 mM ATP, likely degrading to ADP over time; human PFK stocks thus contributed 10–40 µM extra nucleotide (ATP or ADP) to the assay. Each reaction was therefore normalised against a control reaction that included PFK isoform sample but not the exogenous substrate.

**Table 3:** PFK-L has the lowest affinity for F16BP in the reverse reaction Mean average values with SEM values in brackets; *n* = 3.

	*V*_max_ (µmol/min mg)	$K_{0.5}^{ADP}$ (µM)	$K_{0.5}^{F16BP}$ (µM)	*h* (ADP)	*h* (F16BP)
PFK-M	0.63 (0.05)	66.8 (13.4)	804 (66.1)	0.71 (0.22)	1.83 (0.23)
PFK-L	0.62 (0.01)	344.9 (36.4)	2100 (78.4)	1.39 (0.17)	2.49 (0.19)
PFK-P	0.22 (0)	ND	717.8 (58.1)	ND	1.35 (0.12)

All reverse reaction kinetics are consistent with allosteric sigmoidal models both for F16BP titrations and ADP titrations; PFK-M ADP titrations did not show any statistically significant difference between the two models, so the allosteric sigmoidal model was preferred to retain consistency. The reaction obeys allosteric sigmoidal models at ADP concentrations up to 2.5 mM.

PFK-L has low affinity for ADP and F16BP, indicating a reduced propensity for the reverse reaction compared with other isoforms. The sequence identities between the three isoforms range from 68 to 71%, with the ATP-binding site being 85–90% identical (the F6P-binding site has not been fully characterised as yet). It is challenging to interpret these differences in reverse kinetic properties given the lack of precise information about isoform-specific tissue locations, with much of the original published data being measured from relatively crude tissue extracts. However, assuming the original conclusions reached in 1970–1980s [19] are broadly accurate in stating that PFK-L is highly expressed in liver tissues (hence the name: PFK-Liver) then the relative inability of PFK-L to catalyse the reverse reaction may relate to the highly gluconeogenic — and FBPase rich — environment in the human liver. The relatively low activity of PFK-P was also observed in the forward reaction (not shown) and may be an intrinsic property resulting from its susceptibility to time and concentration-dependent inactivation probably caused by dissociation of the active tetrameric form [20].

### Substrate inhibition of the reverse PFK reaction in human PFK isoforms

**Figure 5.**
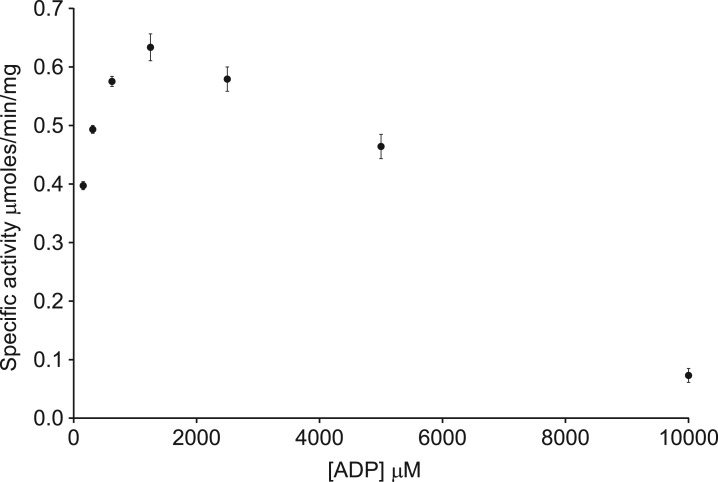
High concentrations of ADP inhibit the reverse reaction by human PFK-M. High concentrations of ADP inhibit the reverse reaction for PFK-M. (Error bars are standard deviations; *n* = 3.)

There is an inhibitory effect at higher concentrations of ADP (above 2 mM) for PFK-M, but the effect is not as clearly demonstrated for the other isoforms (Figure 4A). This effect can be seen more clearly when higher concentrations of ADP are investigated (Figure 5). A similar effect is observed at higher concentrations of F16BP (above 5 mM) for PFK-M (Figure 4B). Standard Michaelis–Menten or allosteric sigmoidal kinetic models should be used with caution when substrate inhibition is present; however, more complex models did not increase the accuracy of data fitting. It is likely that substrate inhibition of the reverse PFK reaction is also present in PFK-L and PFK-P at higher substrate concentrations than were used experimentally but to varying degrees, in a similar way that ATP-dependent inhibition differentially affects the forward reaction in each isoform [21]. The rationale for substrate inhibition of the reverse reaction remains uncertain. It may be analogous to substrate inhibition by ATP in the forward reaction, which enables finer control of the glycolytic flux [22], though it would be surprising if such an effect had been evolutionary advantageous given the presumed small effect on the fitness of precise control of the reverse reaction. High ADP/ATP ratios may enable sufficient ATP production to allow the forward reaction to occur simultaneously; this possibility was reduced — but necessarily eliminated — by adding an excess of GK in the presence of glycerol.

## Discussion

The experiments presented in the present paper provide the first kinetic data on the reverse PFK reaction by eukaryotic ATP-dependent PFKs. The basis for the doctrine that PFK acts only in a forward direction derives from Δ*G*^o^ being highly favourable for the forward reaction.

1 F6P+ATP⇆F16BP+ADP

Δ*G*^o^ = −3.4 kcal mol^−1^ as calculated from the free energies of formation for the products under standard conditions and 1 M concentrations [23]. The free energy of the reaction under different cellular conditions can be estimated from (eqn 2), where the reaction quotient *Q* is the ratio of concentrations of available products and reactants (eqn 3).

2 $$\Delta G=\Delta G^{o}+RT\;\text{ln}\;Q$$

3 Q=[ADP]⋅[F16BP]/[ATP]⋅[F6P]

Estimated cellular concentrations of reactants and products for human and trypanosomes are given in Table 4 and can be used to calculate the reaction quotients (*Q*), the free energy (Δ*G*) and the equilibrium constant (*K*), for the PFK reaction in each of the tissue types. For the reverse PFK reaction (eqn 1) to take place, Δ*G* needs to be greater than 0: (Δ*G*^o^ + *RT*·ln *Q* > 0). Substituting appropriate values (Δ*G*^o^ = −3.4 kcal mol^−1^ and *RT* = 0.543 kcal mol^−1^) we can solve for reaction quotient *Q* ([ADP]·[F16BP]/[ATP]·[F6P]) to show that *Q* must be greater than 500. In other words, a net reverse reaction will only be energetically favourable when the relative concentration of products (F16BP and ADP) is at least 500-fold greater than that of the substrates (F6P and ATP).

**Table 4:** Concentrations of substrates and products of PFK in various mammalian tissues and trypanosomatid glycosomes Δ*G* calculated from eqn (2) using *RT* = 0.543 kcal mol^−1^, where *R* is the gas constant (1.987 cal mol^−1 ^K^−1^) and *T* = temperature (293 K). Equilibrium constant *K* is calculated from *K* = exp (−Δ*G*/*RT*). References for concentrations (directly measured in the non-italicised text, derived from models based on whole cell lysate data in italicised text).

	Erythrocyte	Muscle	Brain	*T. brucei* glycosome
ATP (µM)	1850 [24]	4990 [25]	3325 [30]	*3870* [32]
ADP (µM)	180 [24]	600 [29]	309 [30]	*1315* [32]
F6P (µM)	15.7 [24]	110 [26]	27 [27]	*2400* [31]
F16BP (µM)	7 [24]	32 [28]	200 [27]	*1900* [31]
*Q* (derived)	0.04	0.03	0.69	0.27
*ΔG* (kcal/mol)	−5.09	−5.21	−3.6	−4.7

As shown in Table 4, the concentrations of substrates and products measured in different cell types give estimated reaction quotients of 0.07–0.85 in human tissues and 0.4 in trypanosomatid glycosomes. Additional experimental measurements of muscle from various species, including insects, fish and mammals give ATP/ADP ratios ranging from 11:1 to 1:1 and an average reaction quotient (*Q*) of 0.42 for their PFKs [25]. These low *Q* values show that in most organisms PFK is working far from the reaction's equilibrium. Further experimental studies using mass spectrometry to estimate forward and reverse flux of each step in glycolysis in yeast, *E. coli* and a mammalian kidney cell line [33] identified PFK as an almost exclusively forward driven glycolytic step in all cells. The measured Δ*G* = −3.2 kcal mol^−1^ for PFK in the kidney cell estimated the reverse flux of PFK to be less than 0.7% of the forward flux, with the authors concluding that (under steady-state conditions with cells grown in high glucose media) ‘PFK functions as a classic irreversible step'. Our data show that for human cells, the measured $K_{0.5}^{\mathrm{ADP}}$ values for the reverse reaction (Table 3) exceeds the cytosolic concentrations of ADP (Table 4), but as steady-state concentrations of F16BP are lower than the $K_{0.5}^{F16\mathrm{BP}}$ values, it seems likely that under steady-state conditions the reverse reaction would indeed be inefficient. However, cells can be subjected to extreme conditions of stress and nutrient deprivation and the PFK reverse flux may be very different in non-steady state, glucose-poor conditions and *Q* values of 500 or above could easily be achieved if ATP or G6P were (transiently) depleted.

Despite the apparently high energetic barrier, the reverse reaction for ATP-dependent PFK is possible in cells under certain conditions. Experimentally, the reverse reaction was first shown *in vitro* in *E. coli* PFK at high ADP and F16BP concentrations [34]. Kinetic parameters were determined as $K_{M}^{F16\mathrm{BP}}$ of 398 μM at 2 mM ADP and K_M_^ADP^ 50 µM at saturating concentrations of F16BP. The kinetic parameters suggest generally tighter F16BP and ADP binding compared with the PFK isoforms presented here (Tables 2 and 3), but are not far removed from the kinetic values for PFK-M and this leaves open the question whether there are ever cellular conditions when the reverse reaction could occur.

Metabolite concentrations vary widely between individuals [35] and these differences will be exaggerated further under conditions of metabolic stress. Low concentrations of aldehyde severely deplete ATP levels [36]; cells infected with viruses [37] and necrotic cancer cells [38] have also shown large variations in ADP:ATP ratios. Cells also undergo programmed responses when adapting to new states invoked by events including cell division, apoptosis or nutritional stress which involve concerted changes in activities for families of enzymes (so-called ‘allostatic changes’) [39]. The transition between these states frequently necessitates large swings in metabolite concentrations. One such example is the transition from glycolysis to gluconeogenesis triggered by large changes in the ATP:ADP ratio. There will be a lag period while waiting for up-regulation of FBPase (the gluconeogenic protein that carries out the reverse PFK reaction but not coupled to ATP formation) by increasing transcription/translation or by post-translational modification. During this lag period, we postulate that substrate concentrations may be sufficiently perturbed to permit PFK reversal, before FBPase activity becomes available. The potential role of the PFK filament assemblies [40] and the existence of the as yet poorly characterised glycosome complex [41] or G-bodies [42] that tunnel substrates with potentially very high effective concentrations also provide mechanisms for achieving non-equilibrium concentrations of substrates sufficient to push PFK into reverse.

Trypanosomatids have a uniquely interesting way of organising the glycolytic enzymes with the first seven enzymes in the pathway (including PFK) sequestered in glycosomes [43]. In the bloodstream form of *T. brucei*, there can be over 60 of these organelles per cell, comprising ∼5% of the cell volume [44]. Metabolism in the *T. brucei* parasite has been extensively studied and kinetic parameters for the glycolytic enzymes have been incorporated into a sophisticated *in silico*, experimentally validated metabolic model [40,41]. Despite the good predictive properties of such models, it remains difficult to determine the concentrations of individual metabolites. A potential complication in parameterising *in silico* models is caused by compartmentalisation in eukaryotic cells, as metabolite concentrations may vary significantly between different types of vesicles.

An experimental indication that the reverse PFK reaction may be physiologically relevant and play a role in gluconeogenesis in trypanosomes comes from ^13^C labelled glucose LC–MS work in bloodstream forms of *T. brucei* [45]. This showed that hexose phosphates can indeed be derived (albeit at a low rate of 2%) from F16BP despite no active form of FBPase being detectable in glycosomes, thus possibly indicating reversal of the normal PFK reaction direction. Further biochemical studies suggest that gluconeogenesis occurs after complete knockout of FBPase (F. Bringaud, unpublished) and leaves open the possibility that the reverse PFK reaction may contribute to this pathway. Gluconeogenesis is also carried out in *Leishmania*, but recent studies [46] showed that *Leishmania* amastigotes in activated macrophages cannot use amino acids, instead relying on glycolysis. It is unlikely that FBPase is active in amastigotes *in vivo*, because as this would lead to ATP loss by futile cycling. A likely scenario is that these leishmanial forms have an inactivated form of FBPase (e.g. by post-translational modification), and like the bloodstream-form *T. brucei* in the presence of abundant glucose, PFK may function in reverse. As for mammalian PFK, the estimated physiological reaction quotient of 0.4 (Table 4) is far below the required reaction quotient (500) for the reverse direction. Nevertheless, measuring accurate intra-glycosomal substrate concentrations is technically difficult, meaning that the *in vivo* reaction quotient inside glycosomes may be significantly different from whole cell data. An analogous situation occurs with the *T. brucei* glycosomal GK reaction, for which it is known that reversal occurs *in vivo* with a low ATP/ADP ratio in the organelles (as created under anaerobic conditions) in the presence of glycerol 3-phosphate despite the ΔG° being even less favourable than for PFK reversal [47]. Furthermore, mutagenesis experiments revealed structural optimisation of this enzyme for catalysis of the reverse reaction [48].

Our work shows that LmPFK has significantly lower affinities for ADP and F16BP in the reverse reaction compared with the other trypanosomatid PFKs, though it is activated much more by AMP. The rationale for the differences in trypanosomatid PFK kinetic properties may derive from the differing nutritional environments to which each parasite has become adapted. *T. brucei* is exclusively extra-cellular, usually confined to the haemolymphatic circulation and cerebrospinal fluid, whereas *T. cruzi* is both (transiently) bloodstream and intracellular (cytosolic), infecting a wide variety of cells. *Leishmania* parasites are predominantly restricted to macrophage phagolysosomes, where they may become metabolically quiescent resulting in low growth rates [49]. In this energy restricted environment, the low-energy signal of rising AMP levels may be more important in stimulating glycolytic or gluconeogenic activity, accounting for the greater sensitivity of LmPFK to AMP.

The kinetic data for the eukaryotic ATP-dependent PFKs presented in the present paper help provide a more detailed understanding of the controls governing the glycolytic and gluconeogenic pathways. They will also provide useful experimental data to feed into the increasingly detailed computational models describing these pathways in mammals [50] and trypanosomes [51]. The high reaction quotient (*Q*) required for the reverse reaction can in principle be attained when ATP has been depleted (<100 µM) in the presence of physiologically relevant cellular concentrations of ADP, F6P and F16BP. However, for the human isoforms, the measured *K*_0.5_ values for F16BP are higher than the measured cellular concentrations. To suggest any physiological relevance for the human isoforms would require the existence of an ‘apparent concentration’ of F16BP up to 10-fold higher than has been measured; this is potentially achievable by invoking substrate tunnelling or metabolon structures. The kinetic data measured for the trypanosomatid PFKs would (as for the mammalian PFKs) require a significant increase in ADP/ATP ratio for the reverse reaction, though the cellular concentration of F16BP would (unlike the mammalian case) be above the *K*_0.5_ and sufficient to drive the reaction in the opposite direction. Future more detailed metabolomics studies will continue to deliver more precise data on time-dependent and organelle-dependent metabolite concentrations, which will shed more light on the potential physiological relevance of the reverse PFK reaction.

## Abbreviations

CVs: column volumes
F16BP: fructose 1,6-bisphosphate
F26BP: fructose 2,6-bisphosphate
F6P: fructose 6-phosphate
G6PD: glucose-6-phosphate dehydrogenase
GK: glycerol kinase
IMAC: immobilised metal affinity chromatography
IPTG: isopropyl β-d-1-thiogalactopyranoside
LmPFK: *Leishmania infantum* PFK
MWCO: molecular weight cut-off
PFK: phosphofructokinase
PGI: phosphoglucose isomerase
PPi: inorganic pyrophosphate
TCEP: tris(2-carboxyethyl)phosphine
TEA: triethanolamine

## References

[1] Da Silva D., Ausina P., Alencar E.M., Coelho W.S., Zancan P. and Sola-Penna M. (2012) Metformin reverses hexokinase and phosphofructokinase downregulation and intracellular distribution in the heart of diabetic mice. IUBMB Life 64, 766–774 10.1002/iub.1063 22730258

[2] Granchi C. and Minutolo F. (2012) Anticancer agents that counteract tumor glycolysis. ChemMedChem 7, 1318–1350 10.1002/cmdc.201200176 22684868 PMC3516916

[3] Rajeshkumar N.V., Yabuuchi S., Pai S.G., De Oliveira E., Jurre J.J., Rabinowitz J.D. et al. (2017) Treatment of pancreatic cancer patient-derived xenograft panel with metabolic inhibitors reveals efficacy of phenformin. Clin. Cancer Res. 23, 5639–5647 10.1158/1078-0432.CCR-17-1115 28611197 PMC6540110

[4] Brimacombe K.R., Walsh M.J., Liu L., Vásquez-Valdivieso M.G., Morgan H.P., McNae I. et al. (2014) Identification of ML251, a potent inhibitor of *T. brucei* and *T. cruzi* phosphofructokinase. ACS Med. Chem. Lett. 5, 12–17 10.1021/ml400259d 24900769 PMC4027769

[5] Moore S.A., Ronimus R.S., Roberson R.S. and Morgan H.W. (2002) The structure of a pyrophosphate-dependent phosphofructokinase from the Lyme disease spirochete *Borrelia burgdorferi*. Structure 10, 659–671 10.1016/S0969-2126(02)00760-8 12015149

[6] Chi A. and Kemp R.G. (2000) The primordial high energy compound: ATP or inorganic pyrophosphate? J. Biol. Chem. 275, 35677–35679 10.1074/jbc.C000581200 11001940

[7] Michels P.A., Chevalier N., Opperdoes F.R., Rider M.H. and Rigden D.J. (1997) The glycosomal ATP-dependent phosphofructokinase of *Trypanosoma brucei* must have evolved from an ancestral pyrophosphate-dependent enzyme. Eur. J. Biochem. 250, 698–704 10.1111/j.1432-1033.1997.00698.x 9461292

[8] McNae I.W., Martinez-Oyanedel J., Keillor J.W., Michels P.A.M., Fothergill-Gilmore L.A. and Walkinshaw M.D. (2009) The crystal structure of ATP-bound phosphofructokinase from *Trypanosoma brucei* reveals conformational transitions different from those of other phosphofructokinases. J. Mol. Biol. 385, 1519–1533 10.1016/j.jmb.2008.11.047 19084537

[9] Mertens E., De Jonckheere J. and Van Schaftingen E. (1993) Pyrophosphate-dependent phosphofructokinase from the amoeba *Naegleria fowleri*, an AMP-sensitive enzyme. Biochem. J. 292, 797–803 10.1042/bj2920797 8391256 PMC1134184

[10] Poorman R.A., Randolph A., Kemp R.G. and Heinrikson R.L. (1984) Evolution of phosphofructokinase–Gene duplication and creation of new effector sites. Nature 309, 467–469 10.1038/309467a0 6233492

[11] Bapteste E., Moreira D. and Philippe H. (2003) Rampant horizontal gene transfer and phospho-donor change in the evolution of the phosphofructokinase. Gene 318, 185–191 10.1016/S0378-1119(03)00797-2 14585511

[12] Dunaway G.A., Kasten T.P., Sebo T. and Trapp R. (1988) Analysis of the phosphofructokinase subunits and isoenzymes in human tissues. Biochem. J. 251, 677–683 10.1042/bj2510677 2970843 PMC1149058

[13] Schirmer T. and Evans P.R.P. (1990) Structural basis of the allosteric behaviour of phosphofructokinase. Nature 343, 140–145 10.1038/343140a0 2136935

[14] Blangy D., Buc H. and Monod J. (1968) Kinetics of the allosteric interactions of phosphofructokinase from *Escherichia coli*. J. Mol. Biol. 31, 13–35 10.1016/0022-2836(68)90051-X 4229913

[15] Schöneberg T., Kloos M., Brüser A., Kirchberger J. and Sträter N. (2013) Structure and allosteric regulation of eukaryotic 6-phosphofructokinases. Biol. Chem. 394, 977–993 10.1515/hsz-2013-0130 23729568

[16] Heinisch J. (1986) Construction and physiological characterization of mutants disrupted in the phosphofructokinase genes of *Saccharomyces cerevisiae*. Curr. Genet. 11, 227–234 10.1007/BF00420611 2965996

[17] Berens R. and Marr J. (1977) Phosphofructokinase of *Leishmania donovani* and *Leishmania braziliensis* and its role in glycolysis. J. Protozool. 24, 340–344 10.1111/j.1550-7408.1977.tb00991.x 18601

[18] Mertens E. (1991) Pyrophosphate-dependent phosphofructokinase, an anaerobic glycolytic enzyme? FEBS Lett. 285, 1–5 10.1016/0014-5793(91)80711-B 1648508

[19] Kahn A., Meienhofer M.C., Cottreau D., Lagrange J.L. and Dreyfus J.C. (1979) Phosphofructokinase (PFK) isozymes in man. I. Studies of adult human tissues. Hum. Genet. 48, 93–108 10.1007/BF00273280 156693

[20] Fernandes P.M., Yen L.-H., Kinkead J., McNae I., Michels P. and Walkinshaw M.D. (2017) Effect of ligands and redox state on phosphofructokinase quaternary structure and enzymatic activity. Lancet 389, S36 10.1016/S0140-6736(17)30432-4

[21] Nakajima H., Raben N., Hamaguchi T. and Yamasaki T. (2002) Phosphofructokinase deficiency; past, present and future. Curr. Mol. Med. 2, 197–212 10.2174/1566524024605734 11949936

[22] Meienhofer M.C., Cottreau D., Dreyfus J.C. and Kahn A. (1980) Kinetic properties of human F4 phosphofructokinase. FEBS Lett. 110, 219–222 10.1016/0014-5793(80)80077-9 6445282

[23] Berg J., Tymoczko J. and Stryer L (2001) Biochemistry, 5th edn, pp. 436–437, W.H. Freeman & Co Ltd, New York

[24] Minakami S. and Yoshikawa H. (1966) Studies on erythrocyte glycolysis. II. Free energy changes and rate limitings steps in erythrocyte glycolysis. J. Biochem. 59, 139–144 PMID:4223318 10.1093/oxfordjournals.jbchem.a128274

[25] I. Beis and Newsholme E.A. (1975) The contents of adenine nucleotides, phosphagens and some glycolytic intermediates in resting muscles from vertebrates and invertebrates. Biochem. J. 152, 23–32 10.1042/bj1520023 1212224 PMC1172435

[26] Zalitis J. and Oliver I.T. (1967) Inhibition of glucose phosphate isomerase by metabolic intermediates of fructose. Biochem. J. 102, 753–759 PMID:16742490 10.1042/bj1020753 PMC1270324

[27] Lowry O.H. and Passonneau J.V. (1964) The relationships between substrates and enzymes of glycolysis in brain. J. Biol. Chem. 239, 31–41 PMID:14114860

[28] Spolter P.D., Adelman R.C. and Weinhouse S. (1965) Distinctive properties of native and carboxypeptidase-treated aldolases of rabbit muscle and liver. J. Biol. Chem. 240, 1327 PMID:14284744

[29] Rao D.R. and Oesper P. (1961) Purification and properties of muscle phosphoglycerate kinase. Biochem. J. 81, 405 PMID:14490315 10.1042/bj0810405 PMC1243354

[30] Hisanaga K., Onodera H. and Kogure K. (1986) Changes in levels of purine and pyrimidine nucleotides during acute hypoxia and recovery in neonatal rat brain. J. Neurochem. 47, 1344–1350 PMID:3020172 10.1111/j.1471-4159.1986.tb00763.x

[31] Bakker B.M., Westerhoff H.V. and Michels P.A.M. (1995) Regulation and control of compartmentalized glycolysis in bloodstream form *Trypanosoma brucei*. J. Bioenerget. Biomembr. 27, 513–525 PMID:8718456 10.1007/BF02110191

[32] Graven P., Tambalo M., Scapozza L. and Perozzo R. (2014) Purine metabolite and energy charge analysis of *Trypanosoma brucei* cells in different growth phases using an optimized ion-pair RP-HPLC/UV for the quantification of adenine and guanine pools. Exp. Parasitol. 141, 28–38 10.1016/j.exppara.2014.03.006 24657574

[33] Park J., Rubin S., Xu Y., Amador-Noguez D., Fan J., Shlomi T. et al. (2016) Metabolite concentrations, fluxes, and free energies imply efficient enzyme usage. Nat. Chem. Biol. 12, 277–294 10.1038/nchembio.2077 PMC4912430 27159581

[34] Auzat I. and Garel J. (1992) pH dependence of the reverse reaction catalyzed by phosphofructokinase I from *Escherichia coli*: Implications for the role of Asp 127. Protein Sci. 1, 254–258 10.1002/pro.5560010207 1304907 PMC2142191

[35] Saude E.J., Adamko D., Rowe B.H., Marrie T. and Sykes B.D (2007) Variation of metabolites in normal human urine. Metabolomics 3, 439–451 10.1007/s11306-007-0091-1

[36] Tiffert T., Garcia-Sancho J. and Lew V. (1984) Irreversible ATP depletion caused by low concentrations of formaledhyde and of calcium-chelator esters in intact human red cells. Biochim. Biophys. Acta 773, 143–156 10.1016/0005-2736(84)90559-5 6428450

[37] Ando T., Imamura H., Suzuki R., Aizaki H., Watanabe T., Wakita T. et al. (2012) Visualization and measurement of ATP levels in living cells replicating hepatitis C virus genome RNA. PLoS Pathog. 8, e1002561 10.1371/journal.ppat.1002561 22396648 PMC3291659

[38] Bradbury D.A., Simmons T.D., Slater K.J. and Crouch S.P.M. (2000) Measurement of the ADP:ATP ratio in human leukaemic cell lines can be used as an indicator of cell viability, necrosis, and apoptosis. J. Immunol. Methods 240, 79–92 10.1016/S0022-1759(00)00178-2 10854603

[39] Bermejo-Nogales A., Nederlof M., Benedito-Palos L., Ballester-Lozano G.F., Folkedal O., Olsen R.E. et al. (2014) Metabolic and transcriptional responses of gilthead sea bream (*Sparus aurata* L.) to environmental stress: new insights in fish mitochondrial phenotyping. Gen. Comp. Endocrinol. 205, 305–315 10.1016/j.ygcen.2014.04.016 24792819

[40] Albert M.-A., Haanstra J.R., Hannaert V., Van Roy J., Opperdoes F.R., Bakker B.M. et al. (2005) Experimental and *in silico* analyses of glycolytic flux control in bloodstream form *Trypanosoma brucei*. J. Biol. Chem. 280, 28306–28315 10.1074/jbc.M502403200 15955817

[41] Bakker B.M., Michels P.A.M., Opperdoes F.R. and Westerhoff H V. (1997) Glycolysis in bloodstream form *Trypanosoma brucei* can be understood in terms of the kinetics of the glycolytic enzymes. J. Biol. Chem. 272, 3207–3215 10.1074/jbc.272.6.3207 9013556

[42] Jin M., Fuller G.G., Han T., Inoki K., Klionsky D.J., Kim J.K. et al. (2017) Glycolytic enzymes coalesce in G bodies under hypoxic stress. Cell Rep. 20, 895–908 10.1016/j.celrep.2017.06.082 28746874 PMC5586494

[43] Bakker B.M., Mensonides F.I.C., Teusink B., van Hoek P., Michels P.A.M. and Westerhoff H.V. (2000) Compartmentation protects trypanosomes from the dangerous design of glycolysis. Proc. Natl Acad. Sci. U.S.A. 97, 2087–2092 10.1073/pnas.030539197 10681445 PMC15758

[44] Tetley L. and Vickerman K. (1991) The glycosomes of trypanosomes: number and distribution as revealed by electron spectroscopic imaging and 3-D reconstruction. J. Microsc. 162, 83–90 10.1111/j.1365-2818.1991.tb03118.x 1870115

[45] Creek D.J., Mazet M., Achcar F., Anderson J., Kim D.H., Kamour R. et al. (2015) Probing the metabolic network in bloodstream-form *Trypanosoma brucei* using untargeted metabolomics with stable isotope labelled glucose. PLoS Pathog. 11, e1004689 10.1371/journal.ppat.1004689 25775470 PMC4361558

[46] Saunders E.C., Naderer T., Chambers J., Landfear S.M. and McConville M.J. (2018) *Leishmania mexicana* can utilize amino acids as major carbon sources in macrophages but not in animal models. Mol. Microbiol. 108, 143–158 10.1111/mmi.13923 29411460 PMC7428322

[47] Hammond D.J., Aman R.A. and Wang C.C. (1985) The role of compartmentation and glycerol kinase in the synthesis of ATP within the glycosome of *Trypanosoma brucei*. J. Biol. Chem. 260, 15646–15654 PMID:2999127

[48] Balogun E.O., Inaoka D.K., Shiba T., Kido Y., Tsuge C., Nara T. et al. (2014) Molecular basis for the reverse reaction of African human trypanosomes glycerol kinase. Mol. Microbiol. 94, 1315–1329 10.1111/mmi.12831 25315291

[49] McConville M.J., Saunders E.C., Kloehn J. and Dagley M.J. (2015) Leishmania carbon metabolism in the macrophage phagolysosome- feast or famine. F1000Res 4, 938 10.12688/f1000research.6724.1 26594352 PMC4648189

[50] Marín-Hernández A., Lõpez-Ramírez S.Y., Del Mazo-Monsalvo I., Gallardo-Pérez J.C., Rodríguez-Enríquez S., Moreno-Sánchez R. et al. (2014) Modeling cancer glycolysis under hypoglycemia, and the role played by the differential expression of glycolytic isoforms. FEBS J. 281, 3325–3345 10.1111/febs.12864 24912776

[51] Achcar F., Fadda A., Haanstra J.R., Kerkhoven E.J., Kim D.H., Leroux A.E. et al. (2014) The silicon trypanosome: a test case of iterative model extension in systems biology. Adv. Microb. Physiol. 64, 115–143 10.1016/B978-0-12-800143-1.00003-8 24797926 PMC4773886

