# Supplementary material for: The kinetic characteristics of human and trypanosomatid phosphofructokinases for the reverse reaction
Source: Biochem J. 2019 Jan 18;476(2):179–91. doi: 10.1042/BCJ20180635 (PMC6340114; doi:10.1042/BCJ20180635)
Supplement: Supplementary Figures [file BCJ-476-179-s1.pdf]

SUPPLEMENTARY FIGURE 1

A) AMP activates TcPFK with respect to ADP titrations (F16BP 10mM). 0mM AMP (solid triangles ▲ with solid line), 0.5mM AMP (open triangles △ with dotted line)

B) AMP activates TbPFK with respect to ADP titrations (F16BP 10mM). 0mM AMP (solid squares ■ with solid line), 0.5mM AMP (open squares □ with dotted line)

C) AMP activates LmPFK with respect to ADP titrations (F16BP 20mM). 0mM AMP (solid circles ● with solid line), 0.5mM AMP (open circles ○ with dotted line)

(n= 2, error bars are standard deviations, results for each enzyme normalised to highest specific activity without AMP present).

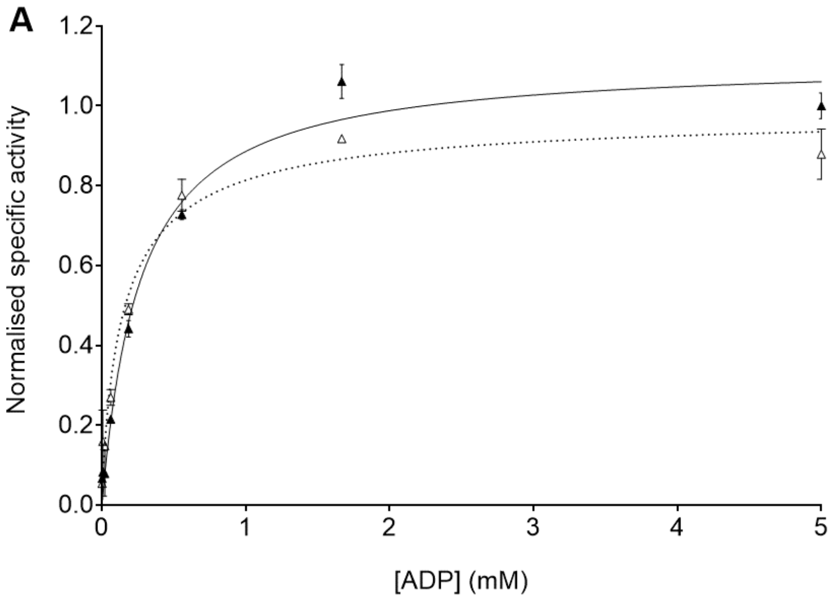

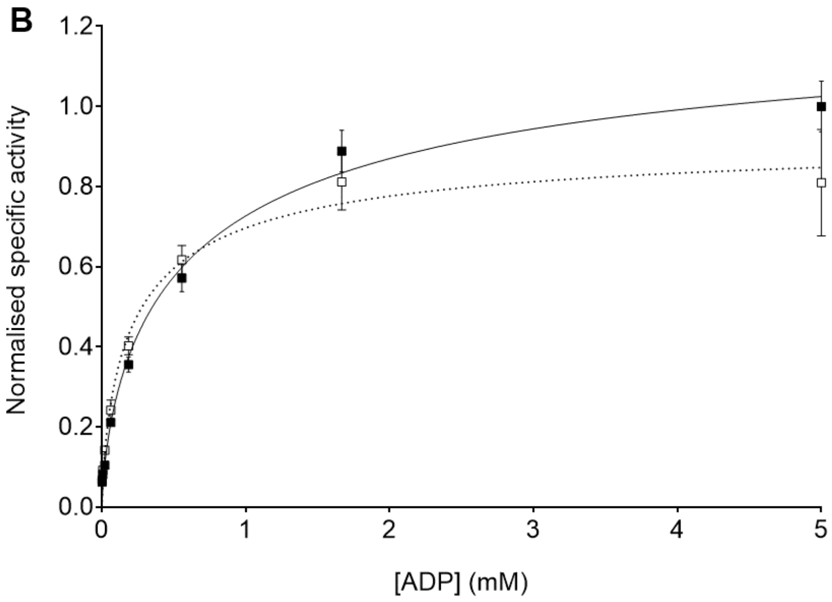

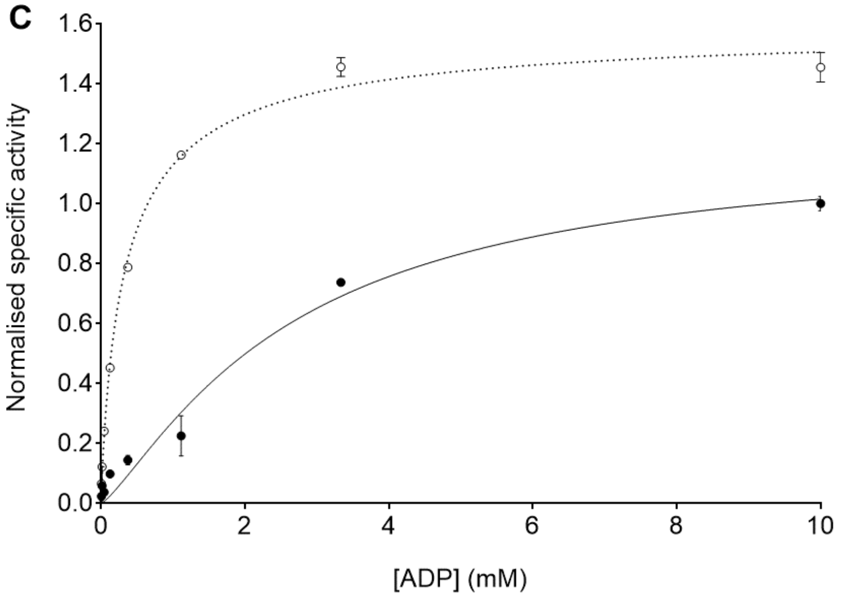
C
1.6
1.4
1.2
1.0
0.8
0.6
0.4
0.2
0.0
Normalised specific activity
0
2
4
6
8
10
[ADP] (mM)

SUPPLEMENTARY FIGURE 2

A) AMP activates TcPFK with respect to F16BP titrations (ADP 5mM). 0mM AMP (solid triangles ▲ with solid line), 0.5mM AMP (open triangles △ with dotted line)

B) AMP activates TbPFK with respect to F16BP titrations (ADP 5mM). 0mM AMP (solid squares ■ with solid line), 0.5mM AMP (open squares □ with dotted line)

C) AMP activates LmPFK with respect to F16BP titrations (ADP 10mM). 0mM AMP (solid circles ● with solid line), 0.5mM AMP (open circles ○ with dotted line)

(n= 2, error bars are standard deviations, results for each enzyme normalised to highest specific activity without AMP present).

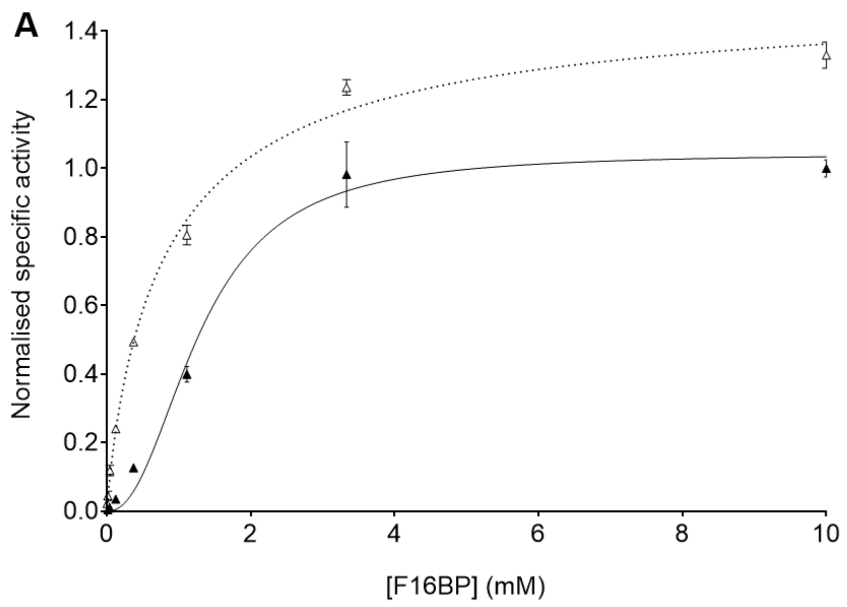

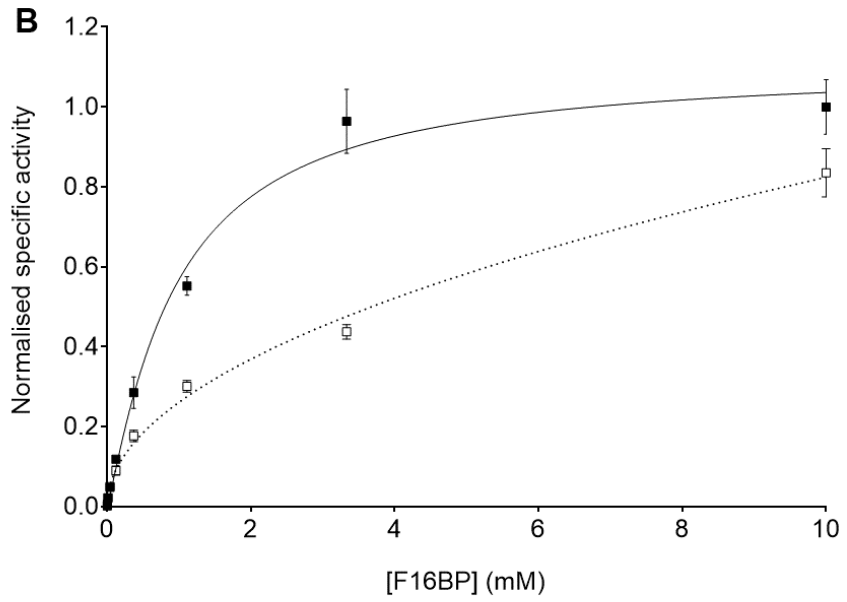

C

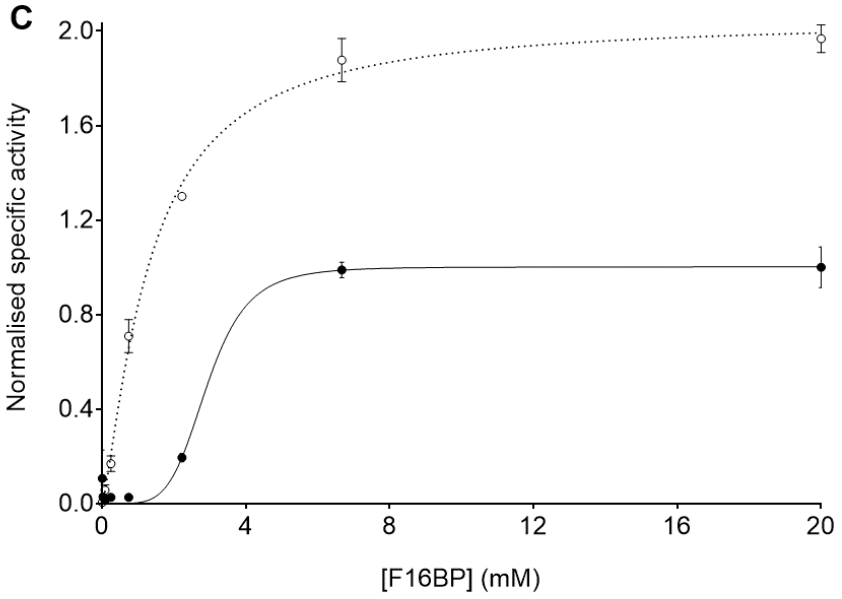

Normalised specific activity
2.0
1.6
1.2
0.8
0.4
0.0
0
4
8
12
16
20
[F16BP] (mM)
